# The use of tranexamic acid for post-tonsillectomy hemorrhage: A scoping review protocol

**DOI:** 10.1371/journal.pone.0319284

**Published:** 2025-02-28

**Authors:** Naomi Tesema, Sukaina Hasnie, Marisa Earley, Max April

**Affiliations:** 1 Division of Pediatric Otolaryngology, Department of Otolaryngology Head and Neck Surgery, New York University School of Medicine, New York, New York, United States of America; 2 Deparmtent of Otolaryngology, Utah Health San Antonio, San Antonio, Texas, United States of America; Universiti Malaya Fakulti Perubatan: University of Malaya Faculty of Medicine, MALAYSIA

## Abstract

**Background:**

Post-tonsillectomy hemorrhage (PTH) is a highly studied outcome of tonsillectomy with serious consequences. Various treatments and interventions have been utilized to decrease post-tonsillectomy hemorrhage. The off-label use of tranexamic acid (TXA) is of growing interest to control PTH but has not been incorporated in management guidelines. This scoping review plans to summarize existing studies from the scientific literature on the use of tranexamic acid for post-tonsillectomy hemorrhage.

**Methods:**

We used the Preferred Reporting Items for Systematic Reviews or Meta-Analysis Extension for Scoping Reviews (PRISMA-ScR). The review will cover studies including patients undergoing tonsillectomy who were treated with TXA in the peri—operative or post-operative period. We include randomized controlled trials, retrospective, prospective, and case series. A database-specific search strategy will be used to search records across. Two reviewers will independently screen and extract data. Tables and visual representations will be utilized to present the extracted data.

**Registration details:**

The protocol will be registered in Open Science Framework and published in PLOS One.

## Introduction

Tonsillectomy ranks among the most performed surgical procedures in the United States, with over 500,000 cases annually involving children under 15 years of age [1]. Though bleeding during the operation is infrequent, post-tonsillectomy hemorrhage (PTH) can occur at rates as high as 7.5% [2]. The peak incidence is between ages 5 and 7 with common indications being recurrent tonsillitis and obstructive sleep apnea [3]. Risk factors for PTH, as revealed by a historical review of a large Welsh population (17,000), include age over 12, male gender, and surgical techniques [4]. Management of PTH may involve observation, gargles, bedside treatment, or a return to the operating room (OR) [2]. Despite affecting one in every twenty tonsillectomy patients, there is a lack of controlled trials addressing its management and no established guidelines or consensus statements [5]. Apart from the risk of secondary PTH, there is a significant burden of costly interventions. Approximately half of the patients presenting to the Emergency Department (ED) with secondary PTH require procedural intervention, roughly doubling the cost of a routine tonsillectomy, in addition to the cost of an ED visit [6]. A survey of pediatric and general otolaryngologists performing tonsillectomies on children revealed that 75% would opt for OR intervention to control PTH, even in the absence of active bleeding at the time of examination [5]. Returning to the OR carries risks such as secondary exposure to anesthetics, heightening neurotoxicity and prolonging postoperative pain, anxiety, and complications [7]. Thus, it would be beneficial to establish effective non-operative treatments to prevent a second surgery. Antifibrinolytics, commonly used in various surgical specialties, including Otolaryngology, can aid in reducing perioperative bleeding [8]. Tranexamic acid (TXA), a synthetic, reversible, competitive inhibitor of the lysine receptor found on plasminogen, prevents the conversion of plasminogen into plasmin, thus maintaining the fibrin clot. Recent studies have demonstrated TXA’s effectiveness in reducing severe epistaxis in the emergency department [9]. However, there is limited knowledge about its use in perioperative tonsillectomy management, especially for children [10]. This lack of information justifies the aim of this scoping review protocol, to comprehensively identify what evidence is available in the existing scientific literature on tranexamic acid use for post-tonsillectomy hemorrhage, identifying research gaps and key trends in usage.

### Review questions

1. What post-operative bleeding outcomes have studies reported on after use of tranexamic acid in pediatric patients undergoing tonsillectomy?
2. In studies of tranexamic acid for tonsillectomy hemorrhage, what are the common modes of tranexamic acid administration in the perioperative and postoperative setting?

## Materials and methods

This review is exempt from approval from the New York University (NYU) Langone Institutional Review Board and will follow the JBI Manuel for Evidence synthesis as a guide on scoping review methods [11]. We used Preferred Reporting Items for Systematic Reviews or Meta-Analysis Extension for Scoping Reviews (PRISMA-ScR) as a guideline for reporting how a scoping review has been conducted [12]. Any changes in the proposed protocol will be edited in the final review.

### Eligibility criteria

The search will include English-language studies involving patients 18 years of age and younger who have undergone tonsillectomy, with any administration of tranexamic acid in the peri-operative or post-operative period. All administration routes will be considered. We will include studies conducted in various settings, such as institution/facility operating rooms and outpatient ambulatory care centers. Excluded are studies focusing on patients with specific bleeding disorders or those not mentioning antifibrinolytic use during or after tonsillectomy surgery. Studies not written in English, describing a surgery other than adenotonsillectomy, or focused on adults will not be included in the review. We are partnering with health science librarians to use Covidence to comprehensively and accurately assess eligibility criteria for the study.

### Information sources and search strategy

A comprehensive literature search across PubMed, Embase, Cumulative Index to Nursing and Allied Health Literature (CINAHL), and Web of Science was conducted from January to April 2024, with all manuscripts uploaded into the Covidence electronic platform. All identified records will be collected and uploaded into Zotero, and duplicates will be removed. Eligible designs include narrative reviews, systematic reviews, cohort studies, case-control studies, and clinical case reports, while non-human studies, non-clinical research, and non-peer-reviewed articles will be excluded. An example of the full electronic search strategy we will use for Pubmed is (tranexamic acid OR TXA) AND (tonsillectomy OR adenotonsillectomy), limited to ‘not adult’ and also limiting to ‘children’). For **Embase:** (tranexamic acid OR TXA) AND (tonsillectomy OR adenotonsillectomy), limited to ‘not adult’ and also limiting to ‘children’). For **CINAHL:** (tranexamic acid OR TXA) AND (tonsillectomy OR adenotonsillectomy), (no limits) For **Web of Science:** (tranexamic acid OR TXA) AND (tonsillectomy OR adenotonsillectomy), (no limits). We will ensure removal of duplicates after totaling the results of these multiple searches.

### Study selection/screening

Two independent reviewers (NT and SH) will screen titles and abstracts based on selection criteria, with discrepancies resolved by MA via Covidence. Subsequently, two independent reviewers (NT and SH) will screen full-text articles against the review’s eligibility criteria. Reasons for excluding full-text articles that do not meet the inclusion criteria will be recorded and reported. Any disagreements between the reviewers will be addressed by discussing with a third reviewer (MA).

### Data charting/collection/extraction

A data charting form will be developed by the team, documenting authors, year, publication type, aims, setting, study design, population demographics, and key findings relevant to the review questions.

### Synthesis and presentation of results

Data will be cleaned using Microsoft Excel and Stata version 6, with a PRISMA flow diagram illustrating the review process. Descriptive statistics aligned with research objectives will be performed, with results presented in tabular format accompanied by a narrative summary.

### Ethics and dissemination

Ethics approval is not required for this review as we are not recruiting patients directly. We plan to disseminate findings through submission to a peer-reviewed journal.

## Discussion

While antifibrinolytics are established in achieving hemostasis across specialties, few studies focus on their use in preventing and managing PTH. Variability exists in the literature regarding administration timing and efficacy in reducing blood loss during tonsillectomy and preventing PTH. Although we used a comprehensive search, it is possible that we could have missed relevant publications because of our search strategy, especially if they were not written in English. This is a limitation of our study that we address with this protocol. This review aims to describe collective findings on PTH outcomes post-tranexamic acid use, informing future studies on PTH treatment. The results will be disseminated to a peer-reviewed journal, and any amendments or changes will be discussed by the group and handled according to recommended guidelines.

## Supporting Information

S1 Checklist PRISMA-ScR Checklist is attached. (PDF)
